# Serum levels of leptin in Nigerian patients with sickle cell anaemia

**DOI:** 10.1186/1471-2326-11-2

**Published:** 2011-05-26

**Authors:** Bamidele A Iwalokun, Senapon O Iwalokun, Semande O Hodonu, Ayoola O Aina, Phillip U Agomo

**Affiliations:** 1 Department of Biochemistry and Nutrition, Nigerian Institute of Medical Research, Yaba, Lagos, Nigeria; 2 IRU PHC, Victoria Island, Lagos, Nigeria; 3 Department of Biochemistry, Lagos State University, PMB 1087, Apapa, Lagos, Nigeria; 4 Maternal and Child Health Complex, General Hospital, Ikorodu. Lagos, Nigeria

**Keywords:** Leptin, Sickle Cell Anaemia, HbSS, Nigerian Patients

## Abstract

**Background:**

Several studies have shown that the pathophysiology of homozygous sickle cell anaemia (SCA) results in a myriad of metabolic, nutritional, haematological and clinical effects that interact with other co-morbid factors to determine the quality of life and life expectancy of afflicted patients. Because of its critical roles in nutrition and metabolism, inflammation, haematopoiesis and cellular immunity, this study determined the plasma levels of leptin in steady and unsteady states of HbSS in Nigerian patients.

**Methods:**

A total of 51 SCA patients aged 5 - 35 years with 34 (61.8%) being females who were either on admission or visiting four medical centres in Lagos, Nigeria together with 22 non-SCD controls aged 5 -30 years comprising 12 (54.5%) females were enrolled after obtaining their informed consent and ethical approval. Patients were further stratified into steady and unsteady cases of SCA based on clinical presentations, while blood samples collected by venipuncture from each of the study participants were analyzed haematologically for full blood count and HbF level and microscopically for malaria, while plasma leptin was assayed using ELISA method. Body composition defined by weight, fat mass and body mass index (BMI) was determined using standard methods. Data obtained for cases and controls were analyzed statistically.

**Results:**

Twenty - one patients had unsteady HbSS and elicited greater and significant (P < 0.05) reduction in fat mass, BMI, HbF and eosinophil count but elevated mean total leukocyte, count, level of irreversibly sickled cells and *P. falciparum *parasitaemia (4613.7 vs. 749.6 - 1078.4 parasites/uL), pyrexia rate (58.3 vs. 25.8%) when compared with steady state patients or non-SCD controls. Compared to the control, significant decreases in plasma leptin before and after controlling for body fat that was worsened by crisis were observed among the SCD patients. Unlike the non-SCD controls, leptin correlated non-significantly (P > 0.05) with all body composition indices measured in the patients except for fat mass in unsteady cases. Multivariate regression analysis identified ESR and RC as independent predictor of low plasma leptin concentration in the SCA patients.

**Conclusions:**

Base on these findings, we conclude that plasma level of leptin is further decreased in the unsteady state of HbSS, shows poor correlation with adiposity and malarial infection but has inflammation and poor reticulocyte response as independent predictors among Nigerian patients.

## Background

Homozygous sickle cell anaemia (SCA) also called HbSS, is a form of sickle cell disease that results from single amino acid substitution (Val for Glu) at the sixth position in each of the two beta globin chain of haemoglobin (α_2_β^s^_2_) and has remained a non-infectious disease of high morbidity and mortality world wide particularly in black populations, Asia and Mediterranean countries [1].

Nigeria has the highest burden of HbSS in the world with ~2% of the population affected by the disease [2]. HbSS produces a myriad of metabolic, haematological, nutritional and clinical effects. Studies have shown that HbSS is associated with decreased food intake, weight gain and delayed puberty especially in females [3].

In the steady state of HbSS, patients are clinically stable but endothelial activation and subclinical vaso-occlusions due to inherent microvascular abnormalities are an ongoing process coupled with chronic but slow red cell haemolysis, platelet activation and moderate cytokine and acute phase response [4]. These pathophysiological events are generally insufficient to induce painful crisis [4,5].

However, in the unsteady state of sickle cell anaemia (SCA), these pathognomic events are exaggerated and the consequence is severe vaso-occlusion and blood flow that leads to organ damage and painful crisis with complications such as bone and joint infarction, acute chest syndrome, severe anaemia, aplastic anaemia and acute renal failure [6]. These complications have well been reported as the causes of hospitalization and deaths in afflicted patients in Nigeria and other countries [7,8].

It has been reported that more than 50% of patients with most severe form of HbSS die before the age of 5 years [8,9]. Studies have also shown that HbSS patients are highly susceptible to infections due to abnormality of their innate immune response including a defective alternate complement system and *Plasmodium falciparum *parasitaemia has been reported by many investigators as a pathognomic factor of painful crisis in patients residing in malaria endemic countries [10,11].

In the past 10 years and with the advent of new technologies and discoveries, there has been an increased interest on ways to increase understanding of pathophysiology of HbSS with regards to the roles played by the regulated inflammatory mediators (e.g. α2 macroglobulin), endothelial stress factors (e.g. soluble thrombomodulin) and platelet factors (e.g. beta TG) in the steady state and how these factors can be modified to improve the quality of life and avert painful crisis in patients [12-14].

Leptin, a 16 kDa peptide hormone, derived mainly from the adipose tissue is a prominent biological factor of energy homeostasis whose circulating levels are a reflection of adiposity in human and animals [15]. The hormone binds to the hypothalamus through the leptin receptor and acts via the JAK/STAT pathway to inhibit the expression norexigenic factors but activate the expression of anorexigenic factors to suppress appetite, food intake and weight gain [16]. Therapeutically, leptin has been found to be beneficial in the management of obesity and lipoatrophy in HIV patients [17], while in wasting and decreased energy intake disease conditions such as cancer, tuberculosis, and hepatitis A, B and C infections, decreased serum levels of leptin were found in consonance with decreased cellular immunity [18-21]. In mice, starvation has been used to reduced serum leptin level and evoke an increased susceptibility to endotoxic shock, while in humans uptake of leptin has been associated with an improved T. cell response and intake of micronutrients [22-24]. Leptin has also been found to play important roles in haematopoiesis as it contributes to erythropoiesis and erythropoietin production by the kidney [25]. There are also evidence that leptin potentiates platelet activation [26].

Given the fact that HbSS patients are inadequate in cellular immunity [7-10], hypercatabolic in metabolism [27], prone to anorexia and growth deficit, elicits higher resting energy expenditure (28) and experience chronic haemolytic and inflammatory episodes even in the steady state relative to non-SCD humans [29], there is a need to understand the possible role of leptin in the pathophysiology of HbSS.

The aim of this study was to determine plasma levels of leptin and investigate its associations with body composition, malaria, haemolytic, sickling and inflammatory parameters.

## Methods

### Study participants

This study analyzed 77 blood samples obtained between December 2009 and May 2010 from SCA patients (n = 55) aged 5 - 35 years (mean age = 15.3 ± 1.1 yr) of whom 30 (54.5%) were males and 22 non-SCD control subjects. The patients were attending sickle cell clinics at Lagos State University Teaching Hospital (LASUTH), Lagos University Teaching Hospital (LUTH), Maternal and Child Complex, General Hospital, Ikorodu, and some district hospitals in Lagos State, Nigeria. To participate in the study, a written informed consent from each patient (directly if adult or through parents/guardian of a child) was obtained. The study protocol was approved by the Ethical committee of the Hospital Management board, Lagos State, Nigeria. SCD-HbSS was ascertained based on a positive sickling test [30] and cellulose acetate electrophoresis of β^s^-haemoglobin at pH 8.6 compared to the controls: HBAA, HBAS, HBSS and HBSC [30]. Patients in a steady state were defined as those without any of the following clinical conditions 4 weeks prior to or at enrollment. They include painful bone crisis, severe anaemia, laboratory diagnosis of bacteremia, acute chest syndrome, aplastic anaemia, splenic sequestration and behaviors such as anxiety and hallucination. Patients with any one or a combination of these clinical and behavioral presentations were said to be in an unsteady state or crisis [1,6,7,29]

### Nutritional Assessment

This was based on the determination of body mass index (BMI) of each study participant. Here, the patients and control subjects were weighed barefoot with minimum clothing using a digital scale. The body weight was recorded to the nearest 0.1 kg. Height was measured to the nearest 0.1 cm using a ruler tape. BMI was then calculated as weight (Kg) divided by the square of height (m^2^). Fat mass (FM) defined as total body weight minus lean mass was determined by calculation [31]

### Laboratory analysis

Blood samples were collected from each study participant by venipuncture into separate and labeled EDTA tubes for haematological and biochemical assays. The haematological parameters assayed include packed cell volume (hematocrit) by the standard heparinized capillary tube method according to Dacie and Lewis [32]. Whole blood haemoglobin level was determined using the Drabkin's reagent [33] and aliquots of blood sample (20 uL each) were lysed in volume ratio 1:20 with Turk's solution (2% glacial acetic acid in water plus gentian violet) and 1% ammonium oxalatae and in ratio 1: 100 with Tommasson solution (NaCl, 1 g; Na_2_SO_4_, 8 g; glycerol, 20 mL; distilled water, 160 mL) for the determination of total leukocyte, platelets and erythrocyte counts. The cells were counted using an improved Neubauer Hematocytometer at × 40 magnification. Differential leukocyte counts of neutrophils, lymphocytes, monocytes, eosinophils and basophils were done by microscopically examined Leishman's stained blood films under oil immersion (× 100 magnification) with results expressed as percentages of the total number of leukocytes enumerated in the high power fields [34]. Absolute count for each leukocyte was then calculated by multiplying the differential count result by the total leukocyte count [29]. Hamatological parameters: mean corpuscular haemoglobin concentration (MCHC) in g/dL of packed cell volume and mean corpuscular haemoglobin (MCH) in pg per cell were also determined by calculation [32]. Erythrocyte sedimentation rate (ESR) in mm/hr was also determined manually using the Western Green method [32]. Reticulocyte counts were performed on blood films pre-stained with brilliant cresyl blue supravital stain for 5 min and expressed as a percentage of 200 nucleated and non-nucletaed erythrocytes counted in 4 - 5 fields. Irreversible sickle cells (ISC) were also examined microscopically (at × 100 magnification) on another Leishman's stained blood film and expressed as a percentage of total number of erythrocytes counted [35]. Thick (~ 12 uL) and thin (3 uL) blood films stained with 4% Giemsa stain for 30 min were also prepared and used for detection and enumeration of malaria parasites. For positive cases, parasites were counted against 200 - 500 leukocytes and expressed as parasites per uL of whole blood by assuming that 1 uL of whole blood contains 8000 leukocytes [36]. A slide was considered to be negative when no parasites were seen after examination of 100 high power fields [36].

Fasted (~ 10 h) plasma recovered from EDTA blood samples by centrifugation at 2500 rpm for 5 min was used for leptin assay. The plasma samples were stored in separate plain tubes at -20°C prior to biochemical analysis. Plasma leptin concentrations were measured by capture ELISA method according to guidelines of the manufacturer (Quantikine DLP00, R&D Systems, Minneapolis, MN). The standard leptin solution was calibrated at 0.5 - 100 ug/L and intra -assay coefficient variation (CV) for low and high controls were 1.3% and 3.0% respectively.

### Statistical analysis

Data expressed as mean ± SD and percentages (%) were analyzed using SPSS version 7.5.2 for Windows (SPSS, Inc., Chicago, IL). Patients and controls were compared regarding their plasma leptin concentrations, body weight, body mass index (BMI) (calculated as weight/height^2^, kg/m^2^) and haematological parameters using Student's t test or Mann-Whitney *U *test as appropriate. Disparity between percentages was evaluated using Chi-square (χ2) test. The relationship between plasma leptin concentrations and BMI and between plasma leptin, fat mass (FM) and each of the haematological parameters measured was analyzed by univariate regression. Multivariate regression models were used to determine independent determinants of plasma leptin levels in SCD-HBSS. Outcomes with probability (P) value < 0.05 were considered to be significant.

## Results

Table 1 presents demographic and haematological characteristics of the sickle cell anaemia (SCA) patients and the non-SCD control. Of the 55 patients enrolled, 31 were steady SCA patients and comprised 16 males and 15 females, while 24 patients were unstable and consisted of 9 males and 15 females. In comparison with the non-SCD control (n = 24), significant (P <0.05) increases in the levels of HbF, ESR, reticulocytes, total leukocytes, neutrophils, lymphocytes, monocytes, eosinophils and platelet counts were found in the SCA patients as a whole. Furthermore, when compared with stable patients, those in unsteady state were found to elicit further significant (P < 0.05) elevations of these parameters including the level of irreversible sickle cells except for HbF and reticulocytes count. The prevalence rates of MCH < 25 pg were found to be 13.6%, 51.6% and 79.2% in the control, steady and unsteady patients respectively (P < 0.05). The percentages of patients with MCHC < 31 g/dL were 9.7% and 29.2% in stable and unstable patients respectively (P <0.05).

**Table 1:** Demographic and Haematological Characteristics of the Sickle Cell Anaemia Patients

		SCA Patients		
Parameter	Control	Total	Steady	Unsteady
No. of cases	22	55	31	24
Sex, Male/Female	10/12	25/30	16/15	9/15
Age, yr	13.0 ± 1.5	15.3 ± 1.1	17.1 ± 1.6*	13.2 ± 1.1
^a^HbF, %	1.3 ± 0.06	3.8 ± 0.2**	4.2 ± 0.2*	3.3 ± 0.2
Hb, g/dL	11.9 ± 0.2	8.3 ± 0.2**	8.9 ± 0.2*	7.4 ± 0.3
PCV, %	35.2 ± 0.5	25.5 ± 0.5**	27.3 ± 0.5*	23.2 ± 0.7
RC, %	1.7 ± 0.1	5.8 ± 0.3**	6.3 ± 0.4*	5.3 ± 0.6
% ICI	ND	6.7 ± 0.3	5.3 ± 0.3*	8.4 ± 0.4
MCHC, g/dL	34.0 ± 0.5	32.4 ± 0.2**	32.8 ± 0.3*	31.7 ± 0.2
MCH, pg	27.4 ± 0.5	23.9 ± 0.4**	25.0 ± 0.4*	22.6 ± 0.6
MCHC < 31 g/dL, n(%)	0(0)	10 (18.2)	3 (9.7)*	7 (29.2)
MCH < 25 pg, n(%)	3 (13.6)	35 (63.6)**	16 (51.6)*	19 (79.2)
ESR, mm/Hr	14.4 ± 3.5	35.1 ± 1.0**	25.7 ± 1.1*	38.4 ± 1.3
WBC, cells/uL × 10^3^	6.1 ± 0.2	11.7 ± 0.1**	11.4 ± 0.2*	12.1 ± 0.1
Neutrophils, cells/uL × 10^3^	3.4 ± 0.1	7.6 ± 0.1**	7.2 ± 0.2*	8.1 ± 0.2
Lymphocytes, cells/uL × 10^3^	2.0 ± 0.09	2.6 ± 0.1**	2.6 ± 0.1	2.4 ± 0.1
Monocytes, cells/uL × 10^3^	0.5 ± 0.04	1.0 ± 0.06**	0.9 ± 0.08	1.2 ± 0.09
Eosinophils, cells/uL × 10^3^	0.2 ± 0.02	0.4 ± 0.03**	0.5 ± 0.04*	0.3 ± 0.04
Basophils, cells/uL × 10^3^	0.08 ± 0.01	0.2 ± 0.02	0.1 ± 0.02	0.2 ± 0.04
Platelet count, cells/uL × 10^3^	241.8 ± 11.6	432.8 ± 82.6**	438.7 ± 11.1*	371.5 ± 16.2
RBC count, cells/uL × 10^6^	4.4 ± 0.07	3.5 ± 0.05**	3.6 ± 0.04*	3.3 ± 0.07

Data are expressed as mean ± SEM or number (%). ^a^HbF was evaluated in 10 control subjects. *P < 0.05 (Steady vs. Unsteady SCA patients); **P < 0.05 (Total SCA patients vs. Control) by Student's t-test or Chi-Square (χ2) analysis; P < 0.05 was considered to be significant.

In Table 2, parasite rates of 13.6%, 19.4% and 41.7% and corresponding densities of 1078.4, 749.6 and 4613.7 parasites/uL were found in the non-SCD controls and SCA patients in steady and unsteady steady are shown. On the whole, the parasite rate and density of the SCA patients were significantly (P <0.05) higher than those of the non-SCD controls. However, the two patient sub-groups did not differ significantly ((P > 0.05) in their parasite rate but showed significant disparity in their level of parasitaemia. The SCA patients were further found to elicit higher mean axillary temperature (37.4 ± 0.07 vs. 37.1 ± 0.04 °C; P <0.05) when compared with non-SCD controls and showed greater increase in this parameter when being in an unsteady state (37.7 ± 0.1 vs. 37.2+0.06 °C). The pyrexia rates in these patients were 25.8% and 58.3% respectively (P < 0.05) (Table 2).

**Table 2:** Levels of *Plasmodium falciparum *parasitaemia among the Sickle Cell Anaemia Patients and non-SCD controls

	SCA Patients			
Parameter	Control	Steady	Unsteady	Total
No. of cases	22	31	24	55
*Pf *parasitaemia, n (%)	3 (13.6)	6 (19.4)	10 (41.7)	16 (29.1)
Relative risk, RR (95% CI)		0.4 (0.08 - 1.7)^		
GMPD, parasites/uL	1078.4	749.6*	4613.7	2335**
Axillary Temperature, (°C)				
Range (mean)	36.8 - 37.4 (37.1 ± 0.04)	36.8 - 37.7(37.2 ± 0.06)*	36.7 - 38. 9(37.7 ± 0.1)	36.7 - 38. 9 (37.4 ± 0.07)**
^@^Pyrexia rate, n (%)	0	6(25.8)*	14 (58.3)	20 (36.4)

Data are expressed as range (mean ± SEM) or number (%); n = Number of positive cases *P < 0.05 (Stable vs. Unstable SCD patients); **P < 0.05 (Total SCD patients vs. Control) by Student's t-test or Chi-Square (χ2) analysis; ^Relative risk was not significant; P < 0.05 was considered to be significant. GMPD = Geometric mean parasite density; ^@^Pyrexia was defined as axillary temperature > 37.4°C

In comparison by gender, significant (P < 0.05) disparity was observed between steady SCA and unsteady SCA patients in the overall mean body weight (26.1 - 31.9 ± 2.9-3.8 vs. 22 - 28.8 ± 2.2 - 2.5 kg) and also specifically in men (40.5 ± 1.0 vs. 33 ± 0.3 kg), girls (19.3 ± 2.8 vs. 17.8 ± 1.4 kg) and women (43.3 ± 3.4 vs. 35.8 ± 0.3 kg) (Table 3). Higher levels of fat mass in boys (3.6 ± 0.5 kg) and women (9.6 ± 0.9 vs. 8.3 ± 0.9 kg); leptin in boys (2.4 ± 0.03 vs. 1.6 ± 0.08 ug/L), girls (4.2 ± 0.2 vs. 2.9 ± 0.1 ug/L) and women (6.9 ± 0.2 vs. 3 ± 0.1 ug/L) and leptin to fat mass ratio (Leptin/FM) in women (0.72 ± 0.05 vs.0.39 ± 0.06 ugL^-1^kg^-1^) were also found in the steady SCA patients compared to unsteady SCA patients (Table 3). Except in boys, the observed disparity in BMI between the two case groups was not significant (P > 0.05) (Table 3)

**Table 3:** Comparative Analysis of Body Composition and Plasma Leptin levels among the Sickle Cell Anaemia Patients in the Steady and Unsteady States

^SCA Patients												
Parameter	Steady Males Boys	Men	Total	Females Girls	Women	Total	Unsteady Males Boys	Men	Total	Females Girls	Women	Total
(n)	(6)	(10)	(16)	(9)	(6)	(15)	(7)	(2)	(9)	(10)	(5)	(15)
Age, yr	8.3 ± 1.4^b^	25.7 ± 1.7^a^	17.1 ± 1.6^a^	8.7 ± 0.8	23.3 ± 2.2	14.5 ± 2.1	10 ± 1.2	17.5 ± 0.5	11.7 ± 1.5	10.9 ± 1.1	20.4 ± 2.2	15.1 ± 1.6
Weight, kg	17.8 ± 1.5^b^	40.5 ± 1.0^a^	31.9 ± 2.9^a,b^	19.3 ± 2.8^a^	43.3 ± 3.4^a^	26.1 ± 3.8^a^	18.8 ± 1.1	33.0 ± 0.2	22.0 ± 2.2	17.8 ± 1.4^c^	35.8 ± 1.3	28.8 ± 2.5
BMI, kg/m^2^	18.4 ± 0.2^a^	19.0 ± 0.3	18.7 ± 0.2	18.9 ± 0.5	19.6 ± 0.8^a^	19.2 ± 0.5	19.7 ± 0.1^c^	18.4 ± 0.4	19.4 ± 0.5	18.5 ± 0.5	17.9 ± 0.3	18.3 ± 0.4
FM, kg	3.6 ± 0.5^a,,b^	8.2 ± 0.4	6.5 ± 0.5	4.6 ± 0.6	9.6 ± 0.9^a^	6.0 ± 0.8	4.8 ± 0.5	8.9 ± 0.04	5.7 ± 0.7	4.4 ± 0.4	8.3 ± 0.9	5.7 ± 0.6
Leptin, ug/L	2.4 ± 0.03^a,b^	2.1 ± 0.05	2.2 ± 0.04^a,b^	4.2 ± 0.2^a^	6.9 ± 0.2^a^	5.3 ± 0.4^a^	1.6 ± 0.08	1.8 ± 0.3	1.6 ± 0.09	2.9 ± 0.1	3.0 ± 0.1	3.0 ± 0.09
Leptin/FM	0.7 ± 0.08^b^	0.27 ± 0.01^b^	0.43 ± 0.06^b^	1.04 ± 0.09^a^	0.72 ± 0.06^a^	0.98 ± 0.08^a^	0.52 ± 0.05	0.2 ± 0.03	0.42 ± 0.05	0.7 ± 0.05^c^	0.39 ± 0.06^c^	0.6 ± 0.05^c^

n = Number of cases, Data are mean ± SEM

^a^P < 0.05 Significantly different in the same gender by age category (Stable vs. Unstable (Mann-Whitney U test)

^b^P < 0.05 Significantly different in the between gender by age category in steady HbSS Patients (Mann-Whitney U test)

^c^P < 0.05 Significantly different in the between gender by age category in unsteady HbSS Patients (Mann-Whitney U test)

^Age was set at ≤ 16 years for boys or girls and > 16 years for men or women

The results presented in Table 4 showed that these parameters were significantly (P < 0.05) higher by gender and age in non-SCD controls compared to SCA patients except weight and BMI. Significant (P < 0.05) disparity in age and fat mass was only observed in male SCA patients (17.1 ± 1.6 vs. 10.8 ± 2 yr) and men (8.4 ± 0.5 vs. 19.3 ± 0.5 kg) respectively (Table 4). Of the anthropometric data correlated with serum leptin level, only age in unsteady SCA patients showed significant (P < 0.05) direct relationship. This was contrary to the significant (P < 0.05) associations between age, body weight, fat mass and serum leptin level seen in the non-SCD control (Results not shown).

**Table 4:** Comparative Analysis of Body Composition and Plasma Leptin levels between the Sickle Cell Anaemia Patients and Control

^Subjects												
			SCA Patients					Control				
Parameter	Males Boys	Men	Total	Females Girls	Women	Total	Males Boys	Men	Total	Females Girls	Women	Total
N	(17)	(8)	(25)	(16)	(14)	(30)	(7)	(3)	(10)	(7)	(5)	(12)
Age, yr	8.3 ± 1.4	25.7 ± 1.7	17.1 ± 1.6^a^	8.7 ± 0.8	23.3 ± 2.2	14.5 ± 2.1	9 ± 0.8	27.2 ± 0.2	10.8 ± 2.0	9.6 ± 1.2	22 ± 2.7	14.8 ± 2.2
Weight, kg	18.2 ± 0.9	35.9 ± 1.2^a,b^	23.9 ± 1.9	17.6 ± 1.6	41.3 ± 1.4	28.7 ± 2.4	18.2 ± 1.7	60.2 ± 1.5	20.8 ± 3.0	21.8 ± 0.8	49 ± 2.6	33 ± 4.4
BMI, kg/mm2	19 ± 0.4	18 ± 0.2^a^	18.7 ± 0.3^a^	18.7 ± 0.4^a^	19.3 ± 0.3^a^	19 ± 0.2	20.1 ± 0.4	23 ± 0.6	20.2 ± 0.4	21.8 ± 0.8	20.7 ± 0.7	21.2 ± 0.4
FM, kg	4.6 ± 0.8	8.4 ± 0.5^a^	5.8 ± 0.5	4 ± 0.3	8.7 ± 0.5	6.2 ± 0.5	4.6 ± 0.5^c^	19.3 ± 0.5^c^	5.5 ± 1.0^c^	6.5 ± 0.7	14.4 ± 0.8	21.3 ± 0.5
Leptin, ug/L	1.9 ± 0.9^a,b^	2.1 ± 0.07	2 ± 0.07^b^	3.7 ± 0.3^a^	2.1 ± 0.7^a^	4.1 ± 0.3^a^	2.5 ± 0.05^c^	2.4 ± 0.09^c^	2.5 ± 0.05^c^	10.1 ± 0.6	16.3 ± 1.4	12.7 ± 1.2
Leptin/FM	0.45 ± 0.03^a,b^	0.26 ± 0.02^a,b^	0.39 ± 0.04^a,b^	0.96 ± 0.09^a^	0.55 ± 0.06^a^	77 ± 0.06^a^	0.58 ± 0.05^c^	0.12 ± 0.004^c^	0.19 ± 0.06^c^	1.6 ± 0.1	1.1 ± 0.1	1.4 ± 0.1

Data are mean ± SEM

^a^P < 0.05 Significantly different in the same gender by age category [SCA Patients vs. Control (Mann-Whitney U test)]

^b^P < 0.05 Significantly different in the between gender by age category in SCA Patients (Mann-Whitney U test)

^c^P < 0.05 Significantly different in the between gender by age category in the Control Patients (Mann-Whitney U test)

^Age was set at ≤ 16 years for boy or girls and > 16 years for men or women

On the effect of haemolysis, inflammation and sickling on leptinemia among the SCA patients, we found non-significant (P > 0.05) association between leptin and haemolytic parameters (PCV, MCHC, MCH, RBC) measured; negative but significant correlation between leptin and ESR (r = -0.55 to -0.79; P <0.01) and between leptin and platelet count (r = -0.39; P = 0.03) only in stable patients. However, serum leptin association with reticulocyte count was positive and significant in stable (r = 0.52; P = 0.003) and all the SCA patients (r = 0.39; P = 0.004) as a whole but not in unsteady state (r = 0.1; P = 0.6). The correlation between serum leptin level and *P. falciparum *parasitaemia was also not significant among SCA patients per subgroups and as a whole (r = 0.11 - 0.32; P >0.05) (Table 5).

**Table 5:** Correlation Analysis on the Relationship between Leptin Levels, malaria, Inflammation, Heamolytic and Sickling parameters among the SCD Patients Studied

SCA Patients						
Variable	Steady (n = 31)		Unsteady (n = 24)		Total (n = 55)	
	r	P	r	P	r	P
**Malaria**						
GMPD.	0.11	0.33	0.35	0.09	0.29	0.1
**Haemolytic**						
PCV	0.28	0.12	0.24	0.32	0.20	0.34
Hb	0.19	0.3	0.22	0.3	0.18	0.38
MCHC	0.15	0.38	0.23	0.27	0.17	0.33
MCH	0.25	0.15	0.24	0.25	0.22	0.29
RBC	0.11	0.56	0.28	0.15	0.14	0.43
**Inflammation**						
ESR	-0.55	0.001*	-0.72	0.000075	-0.59	0.000016*
**Sickling**						
RC	0.52	0.003*	0.1	0.6	0.39	0.004*
ISC	0.32	0.07	0.13	0.55	0.07	0.6
HbF	0.20	0.25	0.16	0.42	0.18	0.37
Platelet count	-0.39	0.03	-0.37	0.08	-0.12	0.72

*Significant P-value (i.e. P < 0.05); r = Correlation coefficient

Furthermore, modeling of the significant variables using multivariate regression analysis revealed reticulocyte count (β= 0.1559; P = 0.03) and ESR (β = -0.082; P = 0.0004) as independent predictor of low leptin level in the SCA patients (Table 6).

**Table 6:** Multivariate analysis of factors associated with Low Leptin Levels in the SCD patients

	Constant	β	[t]	SE	P
	5.2				
ESR		-0.082	4.7	0.017	0.00002*
RC		0.1559	2.3	0.07	0.03*
Platelet count		-0.00083	0.4	0.003	0.7

*Significant P-value (i.e. P < 0.05)

## Discussion

Sickle cell anaemia (SCA) remains a major non-infectious health problem in black populations with Nigeria bearing the greatest brunt of the disease world wide [1,2,8,9]. The results of this study show that haemolytic, sickling and inflammatory episodes coupled with low plasma leptin level before and after correction for fat mass are worsened in Nigeria patients in the unsteady state of SCA.

The low plasma leptin level observed in our SCA cohort agrees with findings of Buchowski [37] who reported a fasting leptin level of 2.26 - 2.36 ng/mL and 5.33 - 7.77 ng/mL in male and female SCD patients relative to 2.49 - 2.59 ng/mL and 15.5 - 28.7 ng/mL in non-SCD male and female controls respectively. After correction for body fat, these workers found that leptin levels in the range of 0.21 -0.74 ng/mL/kg were elicited by both sexes of the SCD patients relative to 0.19 - 1.13 ng/mL/kg by the non-SCD controls. Although similar patterns of reduction in leptin level in SCD patients according to sex were obtained, our observed leptin levels of 2 - 4.1 ng/mL and 2.5 - 12.7 ng/mL for male and female SCD patients are comparatively lower. This disparity might be related to difference in mean ages and sample size of patients used in the two studies. For instance, the mean ages of male and females SCD enrolled in the USA study were 15.4 - 30.6 years and 15.4 - 25.6 years for male and female patients respectively. These ages together with their respective mean weights were higher than those of this study. Although not determined, but considering the geographical divide between the USA study and the present study, dietary pattern of the two enrolled patients may differ and this together with modifiable lifestyle factors have been found to affect the level of leptin in circulation in humans independent of clinical conditions [16,38]. Similar reduction in leptin level has also been found in patients suffering from fulminant hepatic failure, hepatitis A, B and C infections, non-alcoholic steatohepatitis and tuberculosis [19-21,39,40]. To investigate the cause of low plasma leptin level that was further reduced significantly during crisis in our patients, we investigated its relationship with body composition defined by BMI, fat mass. We found no significant correlation between plasma leptin level and both fat mass and BMI, suggesting a possible loss of association between leptin and both adiposity determined by fat mass and BMI in our HbSS cohort. This possibility is further strengthened by our expected finding of positive correlation between leptin and both BMI and fat mass in the non-SCD controls, the observation that the pattern of reduction in plasma leptin levels seen in the two case sub-groups correlated with pattern of reduction in BMI and fat mass by gender and the persistence of lower leptin level in the unsteady SCA sub group after correction for fat mass.

Elsewhere significant positive correlation between leptin and BMI has been found in patients with steatohepatitis, chronic hepatitis B and C infection and acute hepatitis A infections [20,21,40], while van Crevel et al [19] and Buckowski et al [37] reported a positive correlation between leptin and fat mass in tuberculosis and sickle cell disease patients. Leptin has been shown in several studies to elicit positive correlation with acute phase proteins and inflammatory parameters such as c-reactive protein, alpha1-antitrypsin, TNF-α, IL-1 and IL-6 [20] to further show the relevance of this hormone as an indicator of inflammation. In this study mean ESR levels of 25.7 mm/hr and 38.4 mm/hr that were above the normal level (i.e. < 15 m/hr) were found in our steady and unsteady patients cohorts and this confirm chronic inflammatory episode aggravated by crisis as a pathophysiologic component of SCD-HbSS. The higher mean ESR level observed in the unsteady SCA patients might be related to the higher parasite rate and parasitaemia seen in these patients since *Plasmodium falciparum *evoked acute inflammatory response during clinical infection [41]. *P falciparum *infection detected in the control and steady SCA patients can be said to be sub-clinical and hence asymptomatic. Our finding seems to corroborate a study by Kotila *et al *[42] who reported an incidence of asymptomatic malaria in SCA patients from Ibadan south West Nigeria. Malaria is transmitted perennially but with high intensity during the rainy season in Nigeria and 98% of the population is at risk of infections [43]. Furthermore, we found lower asymptomatic parasitaemia level of 749.6 parasites/uL among the steady SCA compared to the parasitaemia level of 1078.4 parasites/uL seen in the non-SCD control and this suggest that high level of parasitaemia may be a trigger of unsteady state or crisis in SCA patients. It also indicates a possible lower fever threshold in the study area being deteriorated by crisis in SCA. The relevance of *P. falciparum *infection to crisis has previously been reported in Nigeria and other SCD population settings where malaria is also endemic [1,2,7], while the observed higher pyrexia rate of 58.3% in our unsteady SCA patients compared with 25.8% in the steady patients further supports the possibility of lower fever threshold that we have suggested. Furthermore, several studies conducted in Nigeria and other endemic countries have attributed susceptibility of SCA patients to clinical and severe malaria to poor complement and antibody responses coupled with the ability to enhance parasite sequestration [4,10].

Since systemic leptin is mainly from the adipose tissue and leptin level rises with increased inflammatory response but decreases with reduced adiposity [15-17], our observed lower plasma leptin level that was exacerbated in an unsteady state in the SCA patients studied represents a balance of these antagonistic events. However, considering the role of leptin in haematopoiesis and effects of adipocyte metabolism on leptin production, the importance of haematological parameters and other adipogenic factors cannot be over-emphasized. The latter was not investigated in the present study, but studies have shown that the levels, uptake or sensitivity to insulin, may influence adipocyte leptin production [44]. With regards to haematological parameters, significantly (P < 0.05) lower level of HbF, a sickling modifier was observed in our unsteady SCA patients compared to their steady counterparts. This suggests that patients in the unsteady state of SCA are poorer in their ability to mount anti-sickling response via HbF and are thus prone to repeated and clinical vaso-occlusions instead of the sub-clinical type, which occurs in a steady state [45].

Under the same shear stress, sickle erythrocytes, owing to their defective cytoskeleton protein-HbS interactions are about 1.5 times and 10 times stickier than normal erythrocyte when oxygenated and during deoxygenation respectively [46]. HbF is known to modify this interaction and its abundance in the erythrocyte has been associated with reduced severity of sickling, vaso-occlusion and clinical manifestations of SCA. This pattern of reduction in HbF level between the steady and unsteady SCA patients studied agrees with previous studies by Ahmed et al [47,48] who reported HbF levels of 6% and 7% in SCD Nigerian patients without and with priapism in one study and found 5.4% and 5% of this parameter in SCA patients without and with opiate dependence in another study. Therefore, our observed HbF levels of 3.3% and 4.2% in this study is suggestive of greater risk of sickling and vaso-oclussions among our cohorts in Lagos. Unlike in Asia, levels of HbF below 10% are common among African SCA patients including Nigeria [45]. Other abnormal haematological parameters observed in this study such as elevated level of irreversibly sickled cells, mean leukocyte, neutrophils, lymphocyte, monocyte, platelets and reticulocyte counts as well as decreased erythrocyte count, PCV, MCH and MCHC are common haematological features of SCA as they have also documented from several studies for SCA patients in Nigeria and other endemic populations [2,47-49]. Mild to moderate leukocytosis is common among SCA patients and this has been attributed to re-distribution of leukocytes from marginal pool to a systemic pool of granulocytes, while moderate lymphocytosis and monocytosis have been implicated as markers of inflammation in SCA patients [1,2,4,47,48]. A study by Wilson *et al *[50] revealed a positive correlation between leptin level and WBC count, while Togo *et al *[51] showed that leptin associates negatively with haemoglobin. In this study, we did not find significant correlation between leptin and these haemotological parameters. This is similar to observations by Caner et al [20] in hepatitis A patients and Koc *et al *[52] in healthy term Japanese infants. Also in this study, the prevalence rates of 29.2% and 79.2% for MCHC < 31 g/dL and MCH < 25 pg were found in our unsteady SCA patients and higher than corresponding rates in steady patients, suggesting higher rate of steady state haemolysis during crisis. Our results are in consonance with similar studies carried out in the northern part of the country and Saudi-Arabia [2,47-49]. It is also important to know that in this study, the reticulocyte and platelet counts of our unsteady SCA patients were found to be lower than levels in steady SCA group. This is contrary to the findings of Ahmed *et al *[48] between opiate dependent and non-dependent SCA patients and between tuberculosis and steady SCA patients. However, our results align well with report by the same group of investigators on SCD patients with and without priapism [47] and the findings of Salawu *et al *[10]. These workers attributed crisis-induced decrease in reticulocyte count to attenuated reticulocyte response to haemolysis in afflicted patients, while crisis-enhanced platelet sequestration was considered to be responsible for their observed platelet reduction. The lowered reticulocyte count in our SCA patients may also be due loss arising from sequestration of the reticulocytes. This is because recticulocytes of SCA patients are more adhesive than those of non-SCD control [2,4,5,7,8] and the results of this study imply that under unsteady state of SCA, the expression and levels of adhesive molecules factors on the surface of reticulocytes could be more. This is however, subject to further investigations.

Elevated platelet has been associated with inflammation characterized by leukocytosis and elevated ESR in various disease conditions including SCD [2,4,47-49] as observed in this study. However, unlike ESR and reticulocyte count, platelet failed as independent predictor of plasma leptin in these patients.

However, this study is limited by the fact that ESR, which was found to be an independent predictor of plasma leptin level is a non-specific marker of inflammation [5] and this may limit its prognostic value in predicting outcome of interventions in SCA patients. Despite this, ESR has been reported as an important inflammatory marker together with other specific markers during steady and unsteady states of SCD [1,2,10]. Nevertheless, there may be a need to investigate other more specific inflammatory markers such as β2-macroglobulin and C-reactive protein in our SCA cohort in subsequent studies to build more confidence of reliance on the less expensive and easy to implement ESR assay. Larger sample size is also needed to ascertaining the fitness of poor reticulocyte response as a determinant of low leptin level in SCA. The fact that the lean mass data in this study was obtained by calculation is itself a limitation as it may bias fat mass data of some patients. However, similar calculation was used for all the patients as previously carried out by Owa and Adejuyigbe [31] in the study area to improve the credibility of the corresponding anthropometric data obtained in this study as much as possible. Future studies on the micronutrient variables such as zinc and vitamin E that have been associated with growth and immunity in SCD patients in relation to serum leptin are also advocated.

## Conclusion

Nevertheless, within the scope of this study, it can be concluded that unsteady state of SCA is associated with much lower plasma leptin level in relation to inflammation and poor reticulocyte response among Nigerian patients.

## Competing interests

The authors declare that they have no competing interests.

## Authors' contributions

BA was responsible for project design, laboratory work and analysis of results, HO took part in the laboratory work, IO and AO put up criteria in the clinical sorting of patients and contribute to interpretation of results and manuscript write up. AU was responsible for project supervision and implementation. All authors have read and approved the final version of the manuscript.

## Pre-publication history

The pre-publication history for this paper can be accessed here:

http://www.biomedcentral.com/1471-2326/11/2/prepub
